# Comparison of Auditory Outcomes between Inpatient- and Outpatient-Based Treatment in Sudden Sensorineural Hearing Loss

**DOI:** 10.3390/jcm11113123

**Published:** 2022-05-31

**Authors:** Hyun-Jin Lee, Yesai Park, Jeon-Mi Lee, Chulyoung Yoon, Tae-Hoon Kong, Eunju Jeon

**Affiliations:** 1 Department of Otorhinolaryngology-Head and Neck Surgery, Incheon St. Mary’s Hospital, College of Medicine, The Catholic University of Korea, Seoul 21431, Korea; idgenesis@naver.com (H.-J.L.); yesai1535@gmail.com (Y.P.); 2 Department of Otorhinolaryngology, Ilsan Paik Hospital, Inje University College of Medicine, Goyang 10380, Korea; entmeowmiya@gmail.com; 3 Department of Biostatistics, Yonsei University Wonju College of Medicine, Wonju 26426, Korea; fezro@yonsei.ac.kr; 4 Department of Otorhinolaryngology-Head and Neck Surgery, Yonsei University Wonju College of Medicine, Wonju 26426, Korea; cochlear84@gmail.com

**Keywords:** sudden sensorineural hearing loss, outpatient-based treatment, inpatient-based treatment

## Abstract

Objective: The primary treatment for sudden hearing loss is high-dose steroid therapy. In some countries, hospitalization has been taken for granted. Although most countries appear to treat sudden hearing loss on an outpatient basis, some other countries have considered hospitalization as necessary. Only a few studies have been conducted on the effect of hospitalization on hearing outcomes. Therefore, we compared the hearing outcome of inpatient- and outpatient-based treatments to determine whether hospitalization affects the recovery of sudden hearing loss. Methods: We conducted a retrospective case review of patients diagnosed with sudden sensorineural hearing loss (SSNHL). In total, 439 patients with SSNHL were enrolled and categorized as either inpatients (group I) or outpatients (group O). Pure-tone audiometry was initially performed before the treatment and 3 months post-treatment. “Recovery” was defined as a hearing gain of 15 dB HL and a final hearing of better than 25 dB. “No recovery” was defined as an improvement of hearing gain of <15 dB 3 months after treatment. To exclude the effect of the level of pretreatment hearing loss, we divided the patients into three subgroups based on their hearing level: <40 dB, 40–70 dB, and >70 dB. To assess the effect of the treatment modality, the patients were divided into three treatment subgroups: systemic steroids (SS), intratympanic steroids (ITS), and a combination of both (SS and ITS). Results: The pretreatment hearing level was significantly higher in group I (61.5 ± 25.4 dB) than in group O (50.3 ± 23.0 dB; *p* < 0.05). The hearing gain was significantly higher in group I (33.3 ± 24.4 dB) than in group O (24.0 ± 21.8 dB; *p* < 0.05). The “Recovery” ratio was significantly higher in group I (70.2%) than in group O (63.1%) (*p* < 0.05). A repeated measures ANOVA was performed to assess the statistical differences between hospitalization, treatment modalities, and pretreatment subgroups. The inpatient group showed a significant hearing improvement in all SSNHL patients (*p* < 0.05). There was a significant hearing improvement in the inpatient group with pretreatment hearing <40 and 40–70 dB (*p* < 0.05). There was no significant difference between the inpatient and outpatient groups in pretreatment hearing >70 dB (*p* > 0.05). Conclusions: This retrospective study showed that inpatient treatment for sudden hearing loss is more beneficial for hearing improvement than outpatient treatment. The positive effect of inpatient treatment appears to be significant in patients with a pretreatment hearing level of 70 dB or less.

## 1. Introduction

In many countries, including Korea, inpatient treatment has been considered helpful for the treatment of sudden sensorineural hearing loss (SSNHL). Therefore, hospitalization-based treatment has been given priority [1,2,3]. Through hospitalization, intensive treatment using various treatment modalities and physical rest can be provided. Furthermore, it is possible to provide comprehensive management, including a low-salt diet and psychological support, tracking any pre-existing systemic disease, and monitoring the potential adverse effects of systemic steroids.

The treatment policy for SSNHL regarding inpatient- or outpatient-based treatment varies between countries. The German treatment guidelines state that the treatment of SSNHL is possible on an outpatient or inpatient basis, but inpatient treatment is recommended for patients with severe SSNHL [4]. In Japan, SSNHL patients are traditionally treated as inpatients [5]. In Taiwan, about half of the patients with SSNHL, particularly those presenting profound hearing loss or severe vertigo, are hospitalized with comprehensive treatment and examinations [6]. In the United States, SSNHL treatment is typically outpatient [1].

In Korea, SSNHL has traditionally been treated with 7 to 10 days of high-dose systemic steroid administration with hospitalization. Medical insurance is provided to the entire nation of Korea, and in-hospital treatment had been available at relatively low prices without special restrictions, which allows for an extended period of hospitalization. Although inpatient treatment is recommended, several patients prefer outpatient treatment. Therefore, we wondered whether there was a difference in the effectiveness of the inpatient and outpatient treatments.

To the best of our knowledge, there are only a few studies on the clinical evidence for the effectiveness of inpatient treatment for sudden hearing loss. Therefore, this study aimed to compare the effects of inpatient- and outpatient-based treatments for sudden hearing loss. 

## 2. Materials and Methods

### 2.1. Subjects

We conducted a retrospective medical chart review of patients diagnosed with SSNHL between 2001 and 2017 in Incheon St. Mary’s Hospital, a tertiary hospital, the Catholic University of Korea. The institutional review board of Incheon St. Mary’s Hospital, the Catholic University of Korea approved this study (OC18RESI0067). In our patient cohort, meticulous history taking, physical and neurological examination, serological tests, and audiometric tests were performed. At the first visit, the tympanic membrane of all patients was examined under a microscope. Sudden hearing loss was diagnosed only when the tympanic membrane was normal, the air-bone gap (ABG) was less than 10 dB on the pure-tone audiometry (PTA), and the tympanogram was type A. Temporal bone computed tomography was performed to observe if there was any suspicion of middle ear disease by microscope examination. Inner ear magnetic resonance imaging was performed in patients with the following signs of suspected retrocochlear pathology: severe vertigo, balance problems, progressive hearing loss, neurological symptoms, low word discrimination score, or abnormal auditory brainstem response [7].

The usual medical practice for SSNHL is inpatient-based therapy in our hospital; if hospitalization was difficult due to various personal reasons, steroids were prescribed, and regular checkups were performed in outpatient clinics. Patients underwent systemic steroid or intratympanic steroid (ITS) therapy. Patients who underwent salvage ITS therapy after systemic steroid treatment were also included. Salvage ITS injection was given four times within a range of 0.3–0.4 mL of dexamethasone solution. The protocol of systemic steroid administration was a 10-day course of oral prednisolone administration: 1 mg/kg/day for the first 5 days and a tapering dose for next the 5 days (0.7 mg/kg/day for 2 days, 0.3 mg/kg/day for 2 days, and 0.2 mg/kg/day for 1 day). The usual maximum dose of oral prednisone was 60 mg/day according to the clinical practice guidelines for sudden hearing loss [7].

This study included patients who (1) met the diagnostic criteria for idiopathic SSNHL [7,8], (2) started steroid treatment within 1 week from the onset, (3) have never received steroid treatment before the beginning of the study, and (4) had completed at least 3 months of follow-up, including PTA. The exclusion criteria were as follows: (1) middle ear disease, (2) bilateral involvement, (3) history of recurrent vertigo, fluctuation of hearing impairment, Meniere’s disease and/or acoustic trauma, and (4) retrocochlear lesions. Of the 418 patients who met the criteria, 233 patients were hospitalized for 5–7 days (group I), while 185 patients were treated on an outpatient basis (group O).

### 2.2. Audiological Analysis

PTA was initially performed before treatment and was repeated 3 months post-treatment. The air- (125–8000 Hz) and bone-conduction thresholds (250–4000 Hz) were measured using pure-tone audiometers in a double-walled audio booth. The mean PTA thresholds for air conduction at 0.5, 1, 2, and 3 kHz (PTA4 = [threshold at 0.5 kHz + 1 kHz + 2 kHz + 3 kHz]/4) were calculated. To determine treatment success, “Recovery” was defined as a hearing gain of 15 dB and a final hearing of better than 25 dB, and “No recovery” was defined as an improvement of a hearing gain of <15 dB HL at 3 months after treatment. To exclude the effect of the level of pretreatment hearing loss on the hearing outcomes, we divided the patients into three subgroups based on the hearing level of < 40 dB, 40–70 dB, and >70 dB. The patients were subdivided according to a previous study that reported that the hearing threshold of 40–70 dB was more responsive to steroid treatment [9]. Hearing outcomes were also compared by dividing the subgroups based on the treatment method: systemic steroids (SSs), intratympanic steroids (ITSs), and a combination of both (SS and ITS).

### 2.3. Statistical Analysis

The results are presented as means ± standard deviation. Continuous variables were compared using the Student’s *t*-test for evaluating differences between unpaired groups. Qualitative variables were compared using crosstabs and Fisher’s exact test. The sphericity test is not possible when there are two repeated measurements. In order to confirm that the correlation between the two repeated measurements is low, a correlation analysis is performed. Since the correlation coefficient was 0.39 as low, a repeated measures analysis of variance (ANOVA) was used as the primary statistical analysis. The 0.05 level was selected for the F significance and 0.80 or better for power analysis. The analysis was conducted using SAS 9.4 (SAS Institute Inc., Cary, NC, USA). For all statistical tests, a *p*-value of <0.05 was considered statistically significant.

## 3. Results

### Patient Characteristics and Treatment Outcomes

The age, sex ratio, affected side, and the duration from onset to initiation of treatment were not significantly different between the two groups. The follow-up period was significantly longer in group I (147.7 ± 94.6 days) than in group O (123.6 ± 76.2 days; *p* < 0.05). Tinnitus, vertigo, diabetes, and hypertension were not significantly different between the two groups (Table 1). The pretreatment hearing level was significantly worse in group I (61.5 ± 25.4 dB) than in group O (50.3 ± 23.0 dB; *p* < 0.05). The final hearing level did not show a significant difference between the two groups (28.3 ± 24.5 dB vs. 25.4 ± 22.5 dB) (*p* > 0.05), which is considered to indicate a positive effect of inpatient treatment (Table 2).

**Figure 1:**
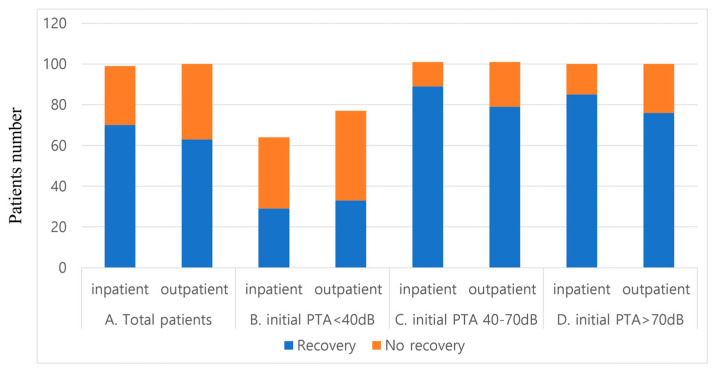
The functional recovery rate based on modified Siegel’s criteria. (**A**) Comparison of the functional recovery rate of all patients (*p* < 0.05). (**B**) Comparison of the functional recovery rate of patients with a hearing level under 40 dB (*p* > 0.05). (**C**) Comparison of the functional recovery rate of patients with a hearing level ranging from 40–70 dB (*p* > 0.05). (**D**). Comparison of the functional recovery rate of patients with a hearing level over 70 dB (*p* > 0.05).

The hearing gain (33.3 ± 24.4 dB vs. 24.0 ± 21.8 dB; *p* < 0.05) as well as “Recovery” rate were also significantly better (70.2% vs. 63.1%; *p* < 0.05) in group I than in group O. (Table 3, Figure 1).

A repeated measures ANOVA was performed to assess the statistical differences between the hospitalization, treatment modalities and pretreatment subgroups. There was no significant interaction among the treatment modalities, hospitalization and pretreatment subgroups (*p* > 0.05). However, a repeated measures ANOVA analysis showed a significant effect between pretreatment subgroups and hospitalization (*p* < 0.05). Pretreatment subgroups and treatment modalities also showed a significant effect (*p* < 0.05). There was no significant interaction between hospitalization and treatment modalities in each pretreatment subgroups (*p* > 0.05) (Table 2). 

Therefore, we compared the pretreatment and final hearing thresholds according to pretreatment subgroups and hospitalization. The inpatient group showed a significant hearing improvement over all SSNHL patients (*p* < 0.05). There was a significant hearing improvement in the inpatient group with pretreatment hearing <40 dB and 40–70 dB (*p* < 0.05). There was no significant difference between the inpatient and outpatient group in pretreatment hearing >70 dB (*p* > 0.05) (Table 3). 

## 4. Discussion

There were more than 500 articles in the literature, after searching PubMed for “sudden sensorineural hearing loss” and “systemic steroid” over the last 5 years, and only four studies addressed admission or outpatient-based treatment. An additional search for “sudden sensorineural hearing loss”, “admission,” and “outpatient” found two more articles (Table 4). Although previous research widely reported the treatment effect of steroids on SSNHL, only a few studies revealed whether inpatient or outpatient treatment was provided. Four studies analyzed hearing outcomes only in patients who had been hospitalized [10,11,12,13]. One study reported the therapeutic effect of sudden hearing loss, noting that all patients received outpatient treatment [14]. To our knowledge, there has been only one study, which compared the effects of inpatient- and outpatient-based treatment in patients with SSNHL [15].

The general advantages of inpatient treatment for sudden hearing loss are as follows: timely and accurate drug administration, monitoring and managing the adverse effects of steroid treatment, and care for accompanying dizziness or underlying diseases (blood pressure, diabetes, etc.). Comprehensive management through hospitalization is expected to have a positive effect on the treatment outcome regardless of the disease. However, it is not yet known to what extent such management helps in the recovery of sudden hearing loss. Kim et al. suggested that rest and relief of social stress and anxiety through inpatient care might help recover the hearing [15]. Most patients experience severe degrees of stress, anxiety, depression, and fear regarding the suddenly developed hearing loss and tinnitus. The extremely negative psychological state can cause neurohumoral dysregulation, which further leads to microcirculation disturbances, such as insufficient blood supply to the inner ear. It is believed that inpatient treatment relieves the physical burden of work and household chores and reduces anxiety through explanation and support from the medical staff. Sun et al. studied the effect of psychological support in hospitalized patients with sudden hearing loss. The patient group that received psychological support showed a higher rate of hearing improvement than the group that received the usual care (85.1% vs. 74.3%) [3].

In terms of final audiograms, group I demonstrated significantly better hearing gain and recovery rates than group O, although group I showed worse pretreatment hearing levels than group O (Table 1, Figure 1). To exclude the effect of the different pretreatment hearing levels of group I, we divided the patients into three subgroups and analyzed the treatment effect separately as follows: patients with a pretreatment hearing loss level of (1) under 40 dB, (2) 40–70 dB, and (3) 70 dB or more. To assess the effect of the treatment modality, the patients were divided into three treatment subgroups: systemic steroids (SS), intratympanic steroids (ITS), and a combination of both (SS and ITS).

When all patients were analyzed, the hearing gain and recovery rates in group I were significantly better than those in group O, although pretreatment hearing was significantly worse than that in group O in our study. As observed in the results presented in Table 1, group I showed poor pretreatment hearing levels, similar to the results of the study by Kim et al. Therefore, the more severe the pretreatment hearing loss, the more likely the patient’s preference for hospitalization because of disease severity.

The study by Kim et al., was the only one that compared the treatment outcomes between inpatient- and outpatient-based treatment. In their study, the disease duration, demographic factors, and comorbidities (vertigo, hearing pattern, and systemic disease) were not significantly different between the two groups (inpatient vs. outpatient). Only the pre-treatment PTA was significantly worse in the inpatient group. Hearing recovery at 3 months post-treatment was compared and categorized into two groups: recovery (≥15 dB of hearing gain) and no recovery (<15 dB of hearing gain). The recovery rate was significantly higher for the inpatient group (52.9%; 129/244) than for the outpatient group (29.8%; 17/57). Through this result, Kim et al. suggested that admission must be strongly recommended for patients with SSHL [15]. Their results were similar to those of our study, in that group I showed better treatment outcomes than group O. The treatment modalities of SS and ITS were mixed in their study; however, each treatment modality was analyzed separately in our study. Considering the treatment modality and pretreatment hearing levels, the final hearing level and hearing gain were better in group I than in group O with the combination treatment in our study. 

However, the proportions of enrolled patients in each group were different. The total number of enrolled patients was similar in both studies, but the patient number in the outpatient group (*n* = 57) was disproportionately small compared with the inpatient group (*n* = 244) in the study by Kim et al. To increase the power of statistical analysis and provide more reliable data, the compared groups must have a matched number of patients. Our study compared two groups with a fairly matched patient population (group I = 233 and group O = 185). 

The duration of time until the initiation of treatment was different in the two studies. Our study population initiated treatment earlier (group I: 3.6 ± 2.4 days and group O: 4.0 ± 2.4 days) than in the study by Kim et al. (inpatient group: 7.1 ± 9.5 days, outpatient group: 6.8 ± 6.4 days). Delay in treatment is one of the negative prognostic factors. For example, Cvorovic et al. [16] reported that significant recovery of hearing dropped to 40% when the patient received treatment more than 7 days from onset, compared with 60% significant recovery in those who received treatment within 7 days from onset. To avoid the negative effect of “delay in treatment,” we excluded the patients whose steroid treatment was delayed for more than 7 days.

Poor prognostic factors of idiopathic SSNHL have been reported as (1) profound hearing loss, (2) down-sloping audiogram shape [17], (3) vertigo, (4) a delay in treatment for more than 10 days, (5) older than 60 years of age, (6) diabetes [8], and (7) hypertension [18,19,20,21,22,23]. Given that hospitalization has long been considered the basis of SSNHL treatment in Korea, doctors have strongly recommended inpatient treatment, especially for patients with poor prognostic factors. Such patients are more likely to consider their condition seriously and accept inpatient treatment. This tendency was also observed in a study by Wu et al. [24] who reported that patients with coexisting vertigo, tinnitus, or diabetes had extended hospital stays. These may have affected the results of the treatment. In addition, systemic diseases are known to affect the outcomes of SSNHL. Wilson [25] observed that diabetic patients with idiopathic SSNHL were less likely to recover their hearing at higher frequencies, and Edizer et al. [22] suggested that hypertension was one of the negative prognostic factors. The presence of vertigo and old age have also been considered as poor prognostic factors [22,23,26]. In our study, there was no significant difference in the proportion of diabetes, hypertension, vertigo, and tinnitus between the two groups.

Several formulas for pure tone average have been employed for clinical or medicolegal purposes: the three-frequency average method (1, 2, and 3 kHz), four-frequency average method (0.5, 1, 2, and 3 kHz), PTAs weighting 1 kHz (0.5, 1, 1, and 2 kHz), or 1 and 2 kHz (0.5, 1, 1, 2, 2, and 4 kHz). Based on Dobie’s research result that the 0.5, 1, 2, and 3 kHz PTA best correlated with communication performance, most tertiary hospitals in Korea use this four-frequency average method (0.5, 1, 2, and 3 kHz) [27,28,29,30,31].

The degree of hearing loss may affect the outcome of steroid therapy. It has been reported that mild hearing loss (<40 dB) was recovered without treatment, and hearing loss of more than 70 dB was not effectively treated; hearing loss of 40–70 dB is known as a “steroid-dependent zone” as a pretreatment hearing level ranging from 40–70 dB shows marked response to steroid treatment [9,21]. The final hearing level and hearing gain in group I were significantly better than in group O for patients with a pretreatment hearing loss of 40–70 dB and under 40 dB in our study. In contrast, in patients with a pretreatment hearing loss of over 70 dB, there was no significant difference in the final hearing level and hearing gain between the groups I and O (Table 3).

When the pretreatment hearing level and treatment modalities were included together, the final hearing and hearing gain were significantly better in group I than in group O with the patients with a pretreatment hearing level of 70 dB or less. Since the treatment modalities did not affect inpatient or outpatient treatment, the results of this study are considered to show a positive effect of inpatient treatment. The limitation of the present study is its retrospective nature, which may have introduced selection bias. A randomized clinical trial is necessary to confirm our result. However, we believe that our research can be of value for the physicians who treat patients with SSNHL and must make decisions regarding the treatment method.

## 5. Conclusions

In this retrospective study, patients who received inpatient treatment for sudden hearing loss with a pretreatment hearing threshold <70 dB showed better hearing outcomes than those who received only outpatient treatment. Additional RCTs are required to support the result of this study.

## Figures and Tables

**Table 1:** Demographic data and treatment outcome of all patients.

Characteristics	Group I (%) (*n* = 233)	Group O (%) (*n* = 185)	*p*-Value
Age (y)	48.7 ± 12.6	50.7 ± 12.7	0.107
Sex (M:F)	110:123	75:110	0.160
Right:left	115:118	76:109	0.061
Duration from onset (day)	3.6 ± 2.4	4.0 ± 2.4	0.08
Follow up period (day)	147.7 ± 94.6	123.6 ± 76.2	0.004 *
Vertigo	65 (27.8)	37 (20.0)	0.086
Tinnitus	158 (67.8)	134 (72.4)	0.062
Diabetes	30 (12.8)	32 (17.2)	0.281
Hypertension	53 (22.7)	42 (22.7)	0.403
Pretreatment hearing level (dB HL)	61.5 ± 25.4	50.3 ± 23.0	0.001 *
Final hearing level (dB HL)	28.3 ± 24.5	25.4 ± 22.5	0.212
Hearing gain (dB HL)	33.3 ± 24.4	24.0 ± 21.8	0.001 *

M: male; F: female. * *p* < 0.05.

**Table 2:** Repeated measures ANOVA for variables.

Variables	*p* Value
Treatment modalities ∗ Inpatient and Outpatient treatment	0.0732
Inpatient and Outpatient treatment ∗ Subgroups	0.0221 *
Treatment modalities ∗ Subgroups	0.0124 *
Treatment modalities ∗ Inpatient and Outpatient treatment ∗ Subgroups	0.2388
Treatment modalities ∗ Inpatient and Outpatient treatment (in Subgroup 1)	0.0912
Treatment modalities ∗ Inpatient and Outpatient treatment (in Subgroup 2)	0.0701
Treatment modalities ∗ Inpatient and Outpatient treatment (in Subgroup 3)	0.9896

Subgroups: subgroups according to pretreatment hearing threshold. Subgroup 1: pretreatment hearing threshold <40 dB, Subgroup 2: pretreatment hearing threshold between 40 to 70 dB, Subgroup 3: pretreatment hearing threshold over 70 dB. (* *p* < 0.05, repeated measures analysis of variance (RM-ANOVA)).

**Table 3:** Comparison of pretreatment and final hearing threshold according to pretreatment subgroups and hospitalization.

	All SSHL Patients (N, Group I = 233, Group O = 185)			Pretreatment Hearing < 40 dB (N, Group I = 61, Group O = 76)			Pretreatment Hearing: 40–70 dB (N, Group I = 80, Group O = 70)			Pretreatment Hearing > 70 dB (N, Group I = 92, Group O = 39)		
	Pretreatment Hearing Threshold (dB)	Final Hearing Threshold (dB)	*p* Value	Pretreatment Hearing Threshold (dB)	Final Hearing Threshold (dB)	*p* Value	Pretreatment Hearing Threshold (dB)	Final Hearing Threshold (dB)	*p* Value	Pretreatment Hearing Threshold (dB)	Final Hearing Threshold (dB)	*p* Value
Group I	60.9 ± 25.6	27.5 ± 23.8	<0.0001 *	28.6 ± 6.9	17.1 ± 13.6	0.0368 *	54.4 ± 9.4	20.3 ± 12.9	0.0021 *	87.9 ± 9.5	40.7 ± 29.8	0.729
Group O	50.0 ± 22.9	25.0 ± 22.1		28.2 ± 7	15.8 ± 8.1		54.3 ± 8.3	26.1 ± 20		84.9 ± 9.2	41.1 ± 32.6	

SSHL: Sudden sensorineural hearing loss, Group I: Inpatient, Group O: Outpatient, N: Patients number (* *p* < 0.05, RM-ANOVA).

**Table 4:** Literature search results related to inpatient or outpatient-based treatment in sudden sensorineural hearing loss.

	Inpatient/ Outpatient	Initiation of Treatment from Onset (Days)	Treatment Protocol	Outcome Measurement	Treatment Assessment Period	Treatment Response
Oh et al., 2007	Inpatient	5.7	10 days of oral prednisolone	Hearing improvement was defined as recovery of more than 10 dB of PTA	Not presented	56.5%
Lim et al., 2012	Outpatient	5.4 ± 3.1	10 days of oral prednisolone	Recovery: –nonserviceable ear: return to serviceable hearing –serviceable ear: improvement of PTA thresholds ≥ 10 dB HL or WRS ≥ 10%	17 days	60.0%
Kim et al.,2015	Inpatient	7	Oral methylprednisolone 80 mg for 4 days, 60 mg for 2 days, 40 mg for 1 day, and tapering over 2 weeks	Siegel’s criteria	3 months	68.8%
Jung et al., 2016	Inpatient	10	10 mg intravenous (iv) dexamethasone for 5 days, and 5 mg iv dexamethasone for 5 days	Siegel’s criteria	3 months	57.7%
Ashtiani et al., 2018	Inpatient	10	Oral prednisolone 75 mg/day over 10 days and Acyclovir for 6 days	10 dB or greater in the mean PTA at five frequencies (0.25, 0.5, 1.0, 2.0, and 4.0 kHz) and improvement of 15% in SDS	4 weeks	60.0%
Kim et al., 2018	Inpatient	7.06 ± 9.53	Oral prednisolone 60 mg for 5 days with dose reductions of 20 mg for every 2 days or dexamethasone (iv) with/without an intratympanic steroid injection (ITS) daily for 6 days	Recovery: ≥15 dB of hearing gain No recovery: <15 dB of hearing gain	3 months	52.9%
	Outpatient	6.84 ± 6.41	Oral prednisolone 60 mg for 5 days with dose reductions of 20 mg for every 2 days or dexamethasone (iv) with/without an ITS twice weekly for six times	Recovery: ≥15 dB of hearing gain No recovery: <15 dB of hearing gain	3 months	29.8%

Repeated measures ANOVA for hospitalization, treatment modalities and pretreatment subgroups.

## Data Availability

The data are available from the corresponding author upon reasonable request.
